# Case report: A successful clinical experience of transplantation of liver and kidney from a donor with myelodysplastic syndromes

**DOI:** 10.3389/fimmu.2024.1360955

**Published:** 2024-04-03

**Authors:** Kang Huang, Qiuyan Zhang, Sanyun Wu, Lihua Zhou, Wenjin Liang, Xiaoyan Hu, Shaojun Ye, Wei Zhou

**Affiliations:** ^1^ Zhongnan Hospital of Wuhan University, Institute of Hepatobiliary Diseases of Wuhan University, Transplant Center of Wuhan University, National Quality Control Center for Donated Organ Procurement, Hubei Key Laboratory of Medical Technology on Transplantation, Hubei Clinical Research Center for Natural Polymer Biological Liver, Hubei Engineering Center of Natural Polymer-based Medical Materials, Wuhan, Hubei, China; ^2^ Department of Ophthalmology, Zhongnan Hospital of Wuhan University, Wuhan, Hubei, China; ^3^ Department of Hematology, Zhongnan Hospital of Wuhan University, Wuhan, Hubei, China

**Keywords:** organ donation, malignancy transmission, myelodysplastic syndromes, organ transplantation, marginal donors

## Abstract

With a shortage of organs for transplant, the use of marginal donors can be an effective measure to meet the shortfall. Myelodysplastic syndromes (MDS) are considered an absolute contraindication for organ donation because of the high invasive potential. Currently, organ transplantation from donors with a past history of MDS has not been reported. In this paper, we report the successful clinical experience of one liver transplantation and two kidney transplantations, with organs donated by a 39-year-old patient diagnosed with a past history of MDS following intracranial hemorrhage. Four and a half years after transplantation, the three recipients are all doing well. However, it is still not clear to what extent organs donated by patients with a past history of MDS can be safely transplanted. This report provides support for the careful use of marginal donors. With effective treatment and full peer assessment, livers and kidneys from donors with a past history of MDS may be safely transplanted.

## Introduction

The demand for organ transplantation worldwide has increased rapidly during the past decade, and the organ shortage crisis has become more immediate. Contraindications for donation have been revised to better meet the demand for organs. The use of lower-quality organs and organs with greater postoperative risk, including organs from older individuals, livers with extensive macrosteatosis, and organs from donors with malignant brain tumors, has become an established routine (1). Relevant evidence linking malignancy transmission from organ donors with a past history of tumors is poor. MDS is a group of very heterogeneous hematological malignancies characterized by inefficient hematopoiesis and a tendency for progression into acute myeloid leukemia (2). To our knowledge, transplantation from a donor with a past history of MDS has not yet been reported. Nevertheless, the incidence of transmission of malignancy remains extremely low. It was reported that the risk of tumor transmission is between 0.01% and 0.05% for each solid organ transplantation (3). A past history of cancer is not always an absolute contraindication for organ donation. Organs may be used when donor malignancy is treated effectively and is in complete remission. Cancer-free period is widely perceived as ‘safe’ period, and the transmission risk of donor-related malignancy is low (4). In this paper, we report the successful clinical experience of one liver transplantation and two kidney transplantations. The organ donor was a 39-year-old woman with a past history of MDS and liver and both kidneys were allocate for transplantation to three recipients. This study aims to provide relevant information for the careful expansion of liver and kidney transplantation from donors with a past history of MDS.

## Case report

### The donor

**Figure 1:**
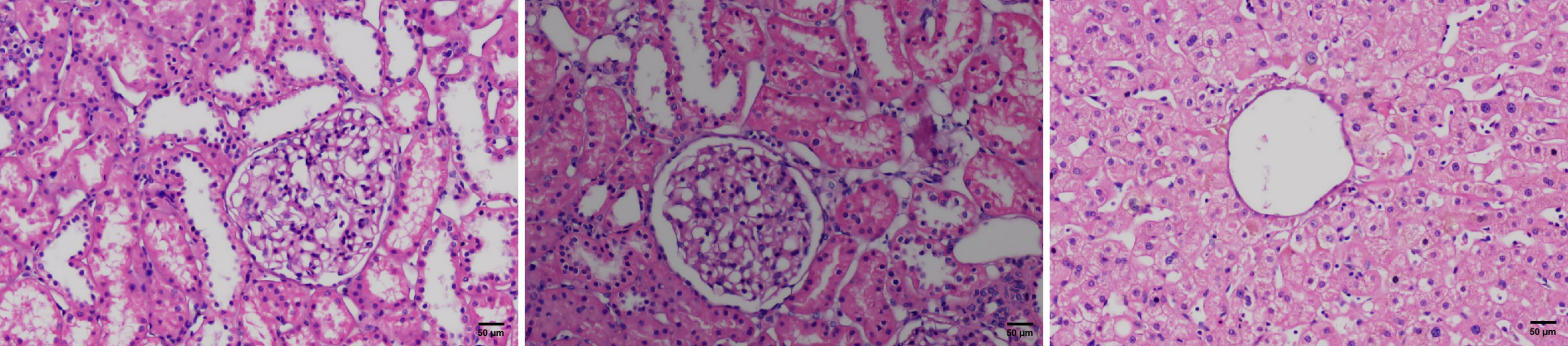
H&E stained microscopic images of the zero-time histology of kidney and liver biopsies from the donor.

In this case, a 39-year-old female donor died from an extensive brain hemorrhage, secondary to cerebral vascular malformation. Her cerebral vascular malformation was not treated. Her medical management included surveillance for infection and related supporting therapy. We take history of disease entirely. She had a prior six-month history of MDS but was in complete remission following treatment, which did not recur during follow-up. She received decitabine 20 mg/m^2^ intravenously daily for 3 consecutive days on 28-day cycle and supportive interventions. After 3 cycles of therapy, she achieved a complete cytogenetic remission. According to a detailed medical history, physical examination, laboratory testing, and computed tomography of brain, chest, and abdomen, there were no aberrant findings or signs of tumor. An expert multidisciplinary team meeting including transplantation, hematology, radiology, histopathology, and oncology was conducted to assess the the risk of donor. Before organ procurement, we collected blood samples and bone marrow samples. The tests suggested the donor was in complete remission, and there were no blast cells in the blood. The following are the donor test results of hemopathy (diagnositics, complete remission and before organ procurement), including cytomorphology of the blood and bone marrrow, cytogenetics, and molecular genetic analyses (**Table 1**). Cranial computed tomography showed large cerebral arteriovenous malformations in the cerebellum. Zero-time histology of kidney and liver biopsies demonstrated no pathological observations (**Figure 1**).

**Table 1:** The laboratory test results of myelodysplastic syndromes in the donor.

Assay	Diagnositics(January 2019 )	Complete remission(April 2019 )	Before organ procurement(June 2019 )
Morphology	BM: blast ratio (2.4%) PH: blast ratio (1.0%)	BM: blast ratio (1.5%) PH: blast ratio (0%)	BM: blast ratio (1.7%) PH: blast ratio (0%)
Flow cytometry	Abnormal flow cytometric immunophenotype	Normal flow cytometric immunophenotype	Normal flow cytometric immunophenotype
Karyotyping	46,XX,del(20)(q11)	Normal	Normal
FISH	D20S108(20q12)	Not detected	Not detected
Fusion gene	WT1 copy number 12462 WT1/ABL ratio 5.617%	WT1 negative	WT1 negative
NGS	ASXL1(NM_015338:exon12:c.C2077T:p.R693X) 67.51% U2AF1(NM_006758:exon2:c.C101T:p.S34F) 42.90%	Not detected	Not detected
Hemoglobin(g/L)	55.2	92.1	107.6
Neutrophil count (×10^9^/L)	1.2	1.9	2.1
Platelets(×10^9^/L)	120	175	246

### Liver recipient

**Figure 2:**
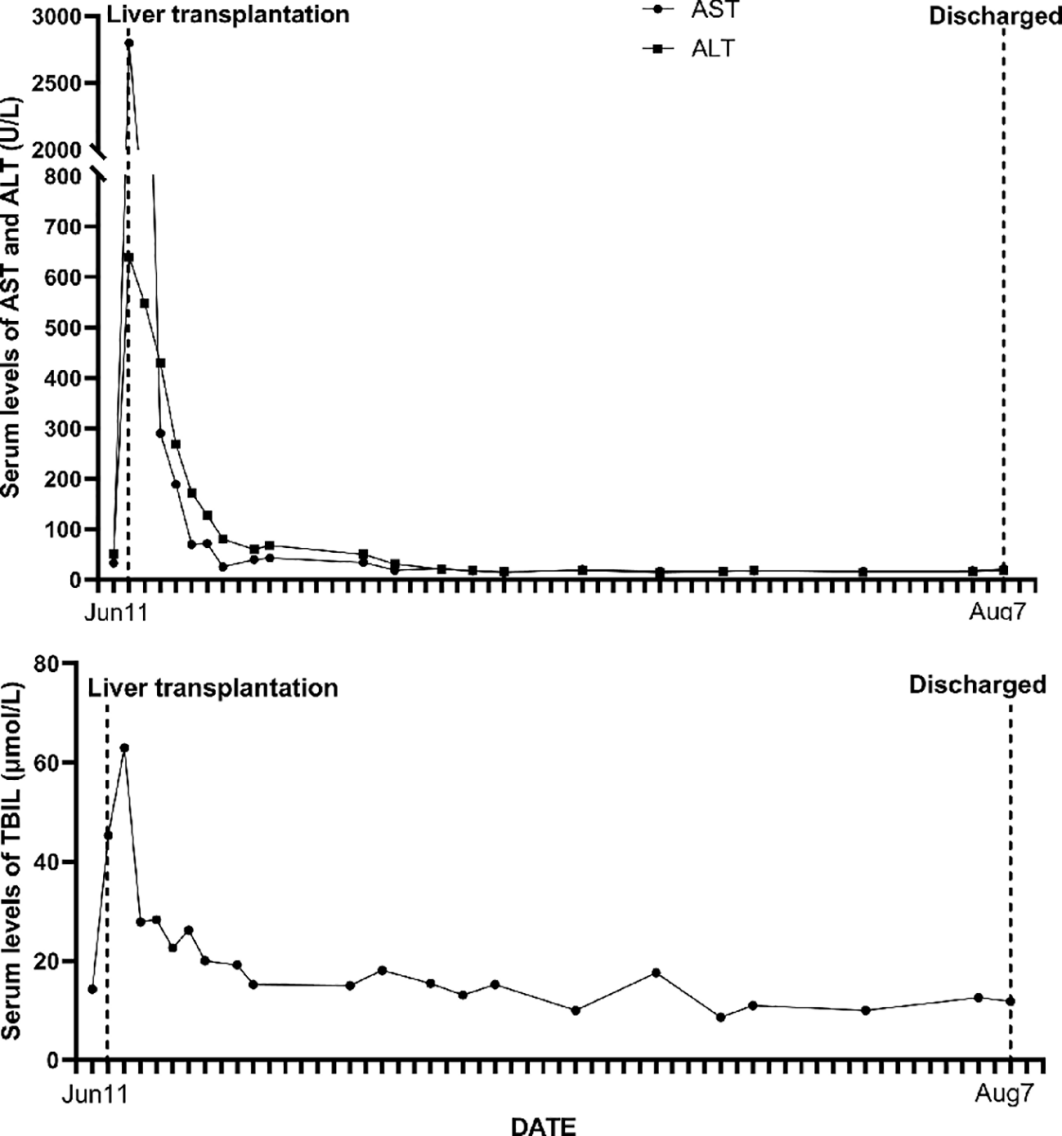
The ALT, AST, and TBIL levels and their dynamic changes in the liver transplant recipient.

The liver was allocated to a 30-year-old male recipient suffering from primary hepatocellular carcinoma. His medical history showed previous health, and he denied hepatitis, tuberculosis, hypertension, diabetes, or heart disease. An abdominal computed tomography (CT) scan revealed a 2.0 × 1.5 cm lesion in the inferior aspect of segment VII in the right hepatic lobe of the liver. His primary hepatocelluar carcinoma was within the Milan criteria and he received a transplant following transarterial chemoembolization. A biopsy of focal liver lesions revealed hepatocellular carcinoma. Hepatitis B surface antigen (HbsAg) and hepatitis B core antibody (HbcAb) were positive. He progressed satisfactorily after orthotopic liver transplant (**Figure 2**). He was treated with an immunosuppression regimen of tacrolimus, sirolimus, mycophenolate mofetil, and methylprednisolone. During the 4.5-year follow-up, hepatocellular carcinoma did not recur and there was no donor-transmitted disease.

### Kidney recipients

**Figure 3:**
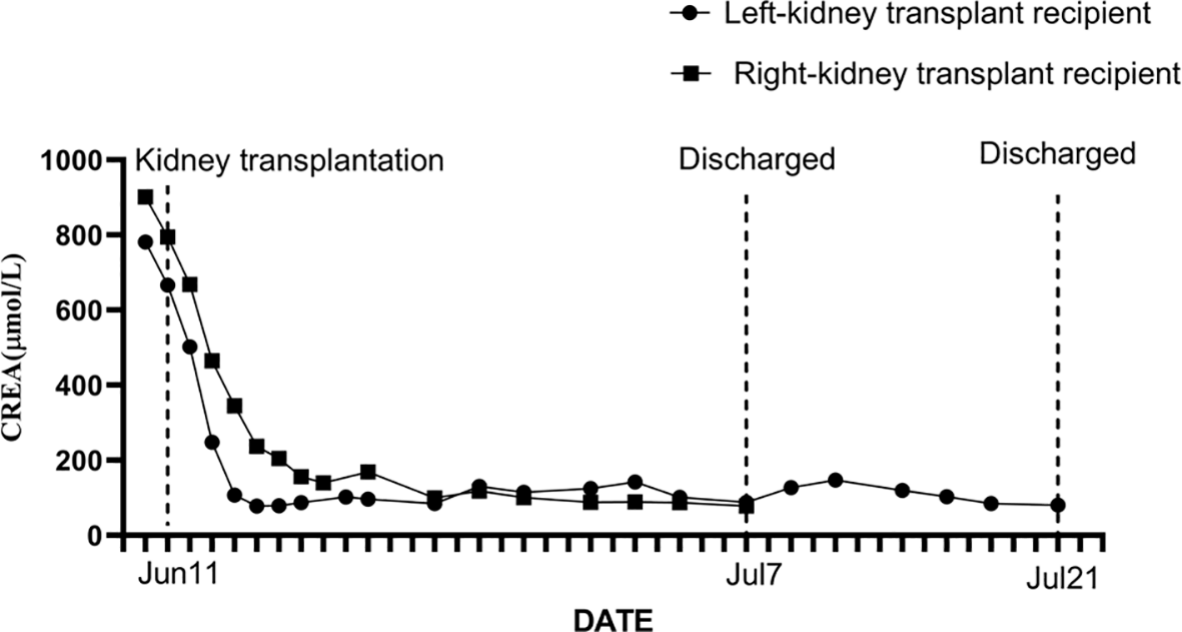
The serum creatinine level and its dynamic changes in the donor’s left-kidney transplant recipient and the donor’s right-kidney transplant recipient.

The left-kidney recipient was a 42-year-old female with stage 5 chronic kidney disease (cryptogenic), who had been receiving regular dialysis for many years. Postoperative recovery was successful and she regained normal kidney function (**Figure 3**). Her immunosuppression regimen consisted of tacrolimus, mycophenolate mofetil, and methylprednisolone; the serum levels of tacrolimus were between 8 and 10 ng/mL. Her renal function (serum creatinine level) and other tests showed no apparent abnormalities at clinical follow-up.

The right kidney was allocated to a 35-year-old male who suffered from end-stage renal disease (cryptogenic). After the transplant, he recovered without complications (**Figure 3**) and regained normal kidney function. He received three rounds of an immunosuppression regimen consisting of tacrolimus, mycophenolate mofetil, and methylprednisolone; the tacrolimus blood concentration was between 8 and 10 ng/mL. He was followed up routinely and no abnormal changes were observed.

## Discussion

Organ shortage is a worldwide problem, and it requires evaluation of all potential donors, including those with a history of malignant disease, depending on the type of malignance, treatment given, and interval time between diagnosis/therapy and organ donation. Prior to this, there has been little high-level evidence to predict the true rate of transmission (5). The US Donor Transmission Advisory Committee has recommended stratifying the risk of tumor types based on the available data of all solid organ transplants and has acknowledged a shortage of strong evidence for malignancy transmission rates by organ (6). Donors with a history of malignancy may be fit for expanding the pool of donors, especially in minimizing potential donor transmitted malignancies. The risk of using organs from donors with a history of cancer needs to be assessed comprehensively before organ procurement. The risk can be reduced by obtaining a complete medical history for a donor, including malignancy types and cancer-free periods in donor selection criteria. It is also necessary to manage multidisciplinary specialist consultations required to clarify the molecular pathological diagnosis and further assess the biological behavior of the cancer (7, 8). In the case reported here, a 39-year-old woman undertook a routine examination and was diagnosed with severe anemia. As a result of further testing, data from bone marrow aspiration and biopsy suggested MDS. After treatment, the patient was in stable condition and showed complete remission during follow-up (**Table 1**). Sometime later, she suddenly fainted and was hospitalized. Brain CT showed an extensive brain hemorrhage due to cerebral vascular malformation. She was seriously ill, and grew worse after treatment. Following assessment of brain death, family consent was given for withdrawal of life support and organ donation. The initial detailed history and physical exam showed this patient had a past history of MDS. Blood and bone marrow tests showed her MDS was in complete remission and there were no blast cells in her blood (**Table 1**). Based on this information, experts in transplantation, hematology, radiology, histopathology, and oncology were invited to evaluate the risk of donation. The gold standard of diagnosis for MDS is cytomorphology of the blood and bone marrrow, supplemented by cytogenetics, histomorphology, and molecular genetic analyses. In the case reported here, the bone marrow of the donor recoverd normal hematopoiesis and there were no blast cells in the blood after treatment (**Table 1**). Karyotype analysis and FISH showed chromosomal abnormality(deletion of the long arm of chromosome 20q11 and 20q12) and chromosomal abnormality did not detect mutation in remission. NGS represents a powerful tool that provides information about mutations driving the disease, the total disease burden and the clonal architecture of haematopoiesis for individual patients (9). And NGS targeted resequencing increases the sensitivity to 1% allete frequency and NGS enables for single base resolution. Over the past decade, the explosion of identified molecular signatures in MDS has been defined, with the development of high throughput sequencing studies (10). The evidence of MDS clone mainly focus on cytogenetic alterations (11). From the results of NGS analysis in this case, ASXL1 and U2AF1 were mutated, and the gene mutation (ASXL1 and U2AF1) did not detect mutation in remission (**Table 1**). All available data suggested that the overall risk of transmission was low. This report highlights the importance of detailed donor assessment, close follow-up, and timely treatment of unexpected donor-transmitted malignancy.

To our knowledge, most transplant surgeons are wary of the use of organs from donors with a risk of disease transmission. Donor-related tumors are an increasingly recognized complication among organ transplant institutions. The incidence of donor-transmitted tumors may rise in parallel with population aging and the use of organs from older donors. Donors with choriocarcinoma, melanoma, breast, colon, and lung cancers appear to carry high risk of recurrence in recipients, while low-grade CNS tumors and skin cancers appear to be at lower risk (12–14). These studies suggest that the Organ Procurement and Transplantation Network(OPTN) data may not fully capture or characterize donor cancer (15). MDS are a heterogeneous group of clonal malignant hematopoietic disorders, characterized by ineffective hematopoiesis in one or more of the lineages of the bone marrow and frequent progression to acute myeloid luekemia (16). Several cases reports have been reported donor cell-derived leukaemias/MDS following haematopoietic cell transplantation (17, 18). However, in the absence of a national analysis of outcomes about donor-transmitted MDS of liver and kidney transplantation, the true risk for donor-transmitted tumors remains unknown. Given the serious shortage of organs for transplantation, such a circumspect attitude may not always be the most appropriate. Current organ shortages mean that patients must endure long waiting time for transplants. It is necessary to balance the risk of using organs from donors where there may be a low or even intermediate risk of disease transmission, against the risk of death in those awaiting transplantation (5, 19). Nalesnik and colleagues helped address this issue and raise awareness of the need to undertake a risk assessment based on the likely benefit for a particular recipient (20). In the case reported here, the liver transplant recipient was a 30-year-old male suffering from primary hepatocellular carcinoma. And he had waited for a liver for one and a half years. Had he not received the transplant, he would probably have missed the optimal timing for liver transplantation. The two kidney transplant recipients had undergone dialysis for many years and had obvious cardiovascular complications. If they had declined the donor kidneys, they might well have waited a long time for another. In the absence of uniform, evidence-based guidelines for the use of an organ from donors with malignancy, such decisions are predominantly based on the physician’s knowledge, experience, and evidence based on case reports and small series. It is, of course, crucial that any potential recipient be warned of the risk that a transplanted organ may transmit malignancy, particularly if it is from a donor with a known history of malignancy. In this case, all available data (blood and bone marrrow, cytogenetics, histomorphology, and molecular genetic analyses) suggested that the donor is in complete remission, widely viewed as ‘safe’ period. The multidisciplinary specialist consultations suggested that the overall risk of transmission was low. It is also important that the recipient is fully informed and closely involved in the decision-making process. The final decision must be based on the principle of maximizing recipient benefits. Additional informed consent are thoroughly informed about the risk of transmittable diseases. Three recipients decided to depend on our expert multidisciplinary team to balance the risk of disease transmission against the risk of death in those awaiting transplantation. And routine follow-up after transplantation, three recipients had not morphological abnormalities and cytopenia in the peripheral blood. Recently, Molecular genetic analyses were conducted on three recipients (**Table 2**). NGS analysis did not detect ASXL1 and U2AF1 mutation in the recipients (**Table 2**). From the results of molecular genetic analyses, it could rule out subclones from the donor to three recipients.

**Table 2:** The laboratory test results in the recipients.

Assay	Liver	Left kidney	Right kidney
Cell morphology of peripheral blood	No blast cells	No blast cells	No blast cells
Flow cytometry	Normal flow cytometric immunophenotype	Normal flow cytometric immunophenotype	Normal flow cytometric immunophenotype
Fusion gene	WT1 negative	WT1 negative	WT1 negative
NGS	Not detected	Not detected	Not detected
Hemoglobin(g/dL)	12.8	12.4	10.3
White blood cell count(×10^9^/L)	7.2	6.0	5.3
Neutrophil count (×10^9^/L)	2.11	3.66	2.84
Platelets(×10^9^/L)	130	194	270

This study aimed to analyze our experience in the use of liver and kidney grafts from donors with a history of MDS. We recommend that the treatment team carefully evaluate the risks and benefits of transplantation given a donor malignancy history, particularly when a potential recipient is in immediate medical need. Ultimately, efforts to expand donor selection criteria must consider the benefit of increased organ availability against the increased mortality associated with poor-quality organs. This study may aid in the expansion of donor selection for those with a history of malignancy, but also suggests the further evaluation of other elements of donor criteria that may be drivers of early mortality.

## Data availability statement

The original contributions presented in the study are included in the article/supplementary materials, further inquiries can be directed to the corresponding author/s.

## Ethics statement

The studies involving humans were approved by Medical Ethics Committee of Zhongnan Hospital of Wuhan University. The studies were conducted in accordance with the local legislation and institutional requirements. The participants provided their written informed consent to participate in this study. Written informed consent was obtained from the individual(s), and minor(s)’ legal guardian/next of kin, for the publication of any potentially identifiable images or data included in this article.

## Author contributions

KH: Writing – original draft, Writing – review & editing. QZ: Data curation, Formal analysis, Investigation, Methodology, Writing – original draft, Writing – review & editing. SY: Data curation, Formal analysis, Investigation, Methodology, Writing – review & editing. SW: Methodology, Writing – review & editing. LZ: Writing – review & editing. WL: Writing – review & editing. XH: Writing – review & editing. WZ: Data curation, Formal analysis, Funding acquisition, Investigation, Methodology, Project administration, Resources, Writing – original draft, Writing – review & editing.
